# High-throughput qRT-PCR validation of blood microRNAs in non-small cell lung cancer

**DOI:** 10.18632/oncotarget.6566

**Published:** 2015-12-11

**Authors:** Petra Leidinger, Thomas Brefort, Christina Backes, Medea Krapp, Valentina Galata, Markus Beier, Jochen Kohlhaas, Hanno Huwer, Eckart Meese, Andreas Keller

**Affiliations:** ^1^ Department of Human Genetics, Saarland University, Homburg, Germany; ^2^ The Comprehensive Biomarker Center GmbH, Heidelberg, Germany; ^3^ Chair for Clinical Bioinformatics, Saarland University, Saarbrücken, Germany

**Keywords:** miRNA, qRT-PCR, NSCLC, diagnosis

## Abstract

Validation of biomarkers is essential to advance the translational process to clinical application. Although there exists an increasing number of reports on small non-coding RNAs (microRNAs) as minimally-invasive markers from blood, serum or plasma, just a limited number is verified in follow-up studies. We used qRT-PCR to evaluate a known miRNA signature measured from blood that allowed for separation between patients with non-small cell lung cancer (NSCLC), COPD and healthy controls.

From the data of our previous microarray studies we selected a panel of 235 miRNAs related to lung cancer and COPD. We observed a high concordance between the AUC values of our initial microarray screening and the qRT-PCR data (correlation of 0.704, *p* < 10^−16^). Overall, 90.3% of markers were successfully validated. Among the top markers that were concordant between both studies we found hsa-miR-20b-5p, hsa-miR-20a-5p, hsa-miR-17-5p, and hsa-miR-106a-5p. The qRT-PCR analysis also confirmed that non-small cell lung cancer patients could be accurately differentiated from unaffected controls: a subset of five markers was sufficient to separate NSCLC patients from unaffected controls with accuracy of 94.5% (specificity and sensitivity of 98% and 91%). Beyond differentiation from controls, we also succeeded in separating NSCLC patients from patients with COPD. MiRNAs that were identified as relevant for the separation between lung cancer and COPD by both qRT-PCR and the array-based studies included hsa-miR-26a-5p, hsa-miR-328-3p and hsa-miR-1224-3p. Although for differentiation between NSCLC patients from COPD patients more markers were required, still high accuracy rates were obtained (5 markers: 78.8%; 10 markers: 83.9%; 50 markers: 87.6%).

## INTRODUCTION

Lung cancer is the leading cause of cancer related death among males in both developed and less developed countries. Lung cancer is estimated to be diagnosed in 224,210 new cases and to sum up to 159,260 deaths in the US [1]. The lack of validated screening procedures leads to an unfavorably late diagnosis of the malignant disease. As seen for a large number of different cancers, detection of lung cancer at an advanced stage results in poor prognosis. Since only 15.4% of lung cancer patients are being diagnosed with the disease still at a localized stage, the 5-year overall survival rate is only 16.8% according to the National Cancer Institute's (NCI) SEER statistics (http://seer.cancer.gov/statfacts/html/lungb.html). There is an urgent need for reliable biomarkers to improve accuracy and time of diagnosis of lung cancer.

MicroRNAs (miRNAs) are small, non-coding RNAs, 17–27 nucleotides in length. They exert their regulatory functions on the expression of multiple genes by initiating translational silencing or degradation of their cognate mRNA targets [2, 3]. Accumulating evidence indicates that miRNA expression patterns are tissue-specific and reflect the (patho-)physiological processes like tumorigenesis, metastasis or drug responsiveness of their cells and tissues of origin [4]. Moreover, miRNAs cannot only be detected in (tumor) tissue but also in blood, serum, urine and other minimal-invasively accessible sources and have the advantage compared to known diagnostic approaches such as low-dose CT that patients are not exposed to radiation. These features make miRNAs promising biomarker candidates for diagnosing, predicting and monitoring diseases like cancer [4–8]. While serum biomarkers might be more suitable for prognosis [9], we and others showed that blood derived miRNA profiles are well suited for detecting diseases in general or also at early stages [7, 10, 11].

We have successfully established a robust assessment pipeline of disease specific miRNA signatures from whole blood samples. Blood borne miRNAs are thought to be valuable biomarkers in that they indicate changes of the immune system in response to a disease development. The miRNA assessment is based on optimized protocols including collection, handling, storage and shipment of miRNA samples. Using respective protocols, we identified accurate disease classifying miRNA signatures from whole blood for neurologic, cardiologic, inflammatory and oncologic diseases [12].

Here, we selected miRNAs towards a blood-borne differentiation between patients with non-small cell lung cancer (NSCLC), patients with chronic obstructive pulmonary diseases (COPD) and unaffected controls. The selection of the miRNAs was based on our previous miRNA microarray studies of patients with lung cancer and COPD [11, 13]. Using Fluidigm qRT-PCR we tested the diagnostic potential of miRNA signatures for lung cancer and COPD.

The primary goal of the study was the validation of the microarray results in a larger and independent cohort using qRT-PCR as technology. Since the translation of research discoveries to clinical care is also a crucial point, we selected a study set-up that facilitates to answer important questions towards this translation. Beyond the validation, we secondly analyzed whether two commonly used miRNA extraction approaches have an influence on the qRT-PCR results. As third aspect we considered the challenge of selecting the best endogenous control in qRT-PCR experiments. All three factors are of high relevance to further the translation of miRNAs to clinics. The identified miRNA panel contributes towards a refined NSCLC miRNA biomarker signature alleviating the need for costly, complex invasive procedures.

## RESULTS

The primary goal of this study is the targeted analysis of a larger miRNA panel identified in our previous microarray studies to define a small panel of miRNAs suitable to separate patients with non-small cell lung cancer (NSCLC), patients with chronic obstructive pulmonary disease (COPD), and unaffected controls. Secondary and tertiary goals were to understand the influence of different miRNA extraction approaches and the usage of various endogenous controls.

### Selection of miRNAs

Based on data of our previous studies, we started with 235 selected miRNAs (details on the 235 miRNAs are provided in Supplementary Table 2). Using the lung miRNA panel consisting of the 235 miRNAs we analyzed 120 individuals by Fluidigm qRT-PCR: 74 NSCLC patients, 26 patients with chronic obstructive pulmonary diseases (COPD) without lung cancer and 20 physiologically unaffected controls. We used five different endogenous controls, including RNU6B, RNU24, RNU44, RNU48 and RPL21. The qRT-PCR of the 120 blood samples was done in triplicate and the results were merged to median values. All measurement results where curated for linear dynamic range of threshold cycle (C_t_) and qPCR curve quality performance as detailed in the Methods section. By applying stringent quality criteria we obtained a set of 128 mature miRNAs that are expressed in blood and can be reliably detected by qRT-PCR. These 128 miRNAs were used for all further analyses.

### Selection of RNA extraction approach

In total we analyzed 120 blood samples. One aspect of the study was to understand the influence of different RNA isolation kits. We used the PAXgene Blood miRNA Kit for RNA isolation of 50 blood samples (10 controls and 40 NSCLC samples) and the miRNeasy Mini Kit for RNA isolation of 70 blood samples (10 controls, 34 NSCLC cancer samples and 26 COPD samples). For both methods we determined the miRNA expression and compared the miRNA levels between NSCLC and controls. Focusing on the C_t_ values we computed a correlation of 0.75 (*p*-value < 10^−15^) between both RNA extraction methods. Considering the AUC of the comparison NSCLC to controls we calculated an even higher correlation of 0.77 (*p*-value < 10^−15^). Considering that we analyzed biological replicates the influence of the extraction approach on the miRNA profiles seems limited. For the comparison between qRT-PCR and microarray results we used only samples that were isolated by miRNeasy Mini Kit.

### Selection of endogenous controls

To identify the most appropriate endogenous control, we tested five commonly used small RNAs including RNU6B, RNU24, RNU44, RNU48 and RPL21, the latter of which is a small protein coding RNA. Highest expressed was RNU48 (average C_t_ of 8, standard deviation of 1.4) followed by RNU44 (average C_t_ of 12, standard deviation of 2.1). We first considered the mean and standard deviation of each control and calculated the coefficient of variation (CV). Based on C_t_ values the endogenous control with lowest coefficient of variation was RNU6B (average C_t_ of 20.9, standard deviation of 2), followed by RNU24. Following transformation (2^−Ct^), RNU48 had the lowest CV again followed by RNU24. The program NormFinder [14] also selected RNU24 and RNU48 as the best endogenous controls.

**Figure 1:**
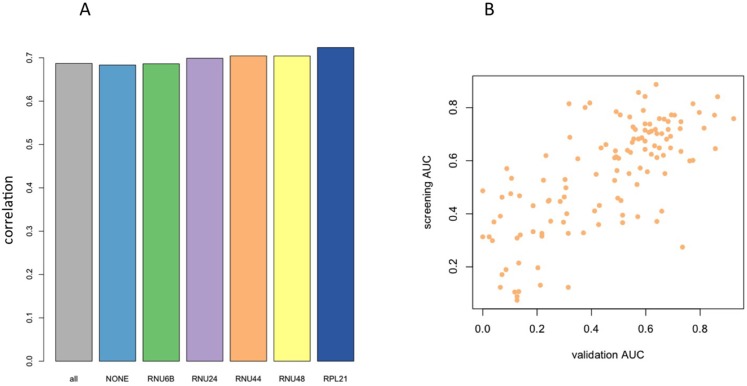
(**A**) Bar Diagrams showing the correlation of qRT-PCR profiles (AUC values) with microarray profiles from the initial discovery study. Different endogenous controls show only minor variability in performance. The first bar (all) represents the correlation obtained by including all ΔC_t_ values, the second bar (none) is calculated based on the raw C_t_ values. (**B**) Scatter plot of AUC values in the microarray discovery study versus the qRT-PCR validation study with respect to RNU44 as endogenous control.

Besides this consideration, we computed the AUC values of each of the 128 miRNAs for differentiating the lung cancer cases from controls based either on raw C_t_ values, or on each of the five different ΔC_t_ values that were obtained by using the different endogenous controls. Then we asked, which of the endogenous controls shows the highest concordance to the initial microarray data. To this end, we first matched the miRNA sequences of the initial study (miRBase v12 annotation and nomenclature) to the most recent miRNA identifiers used in the present study (miRBase v21). The bar diagram in Figure 1A presents the correlation between the raw C_t_ data and the microarray values, and the correlations between each of the five normalized ΔC_t_ values and the microarray values. Thereby, we observed an almost equally well matching between the AUCs of the normalized ΔC_t_ values and the microarray based AUC values as well as the C_t_ values and the initial microarray results. For RNU44, RNU48 and RPL21, correlation exceeded 0.7 (*p* < 10^−16^). The main difference between the endogenous controls lies in their different relative abundance ranging from the lower via the intermediate to the upper end of the dynamic C_t_ range (RNU24, RNU48, RNU44, respectively) providing appropriate normalization for a wide range of different signature miRNA expression levels.

From the analyses in this section we conclude that RNU24 was the most constant control, however being low abundant. The highest correlation to the screening results was obtained for RPL21, which was also low expressed. A reasonable compromise between stability and concordance to microarray results was reached for RNU44 and RNU48. In the following we focused our analysis of RNU44, with respect to this endogenous control the scatter plot between screening and validation is presented in Figure 1B.

### Detailed comparison between screening and validation

To further compare the results of the Fluidigm qRT-PCR validation and our initial microarray findings, we specifically considered the miRNAs that were significant in the initial microarray data set. In order to limit the bias between different miRNA sets we considered the unadjusted *p*-values. According to the previously described results we focused on the ΔC_t_ values with respect to RNU44 as endogenous control. Of the 128 markers, 62 were significant in the screening study. For 56 of those, we found a dys-regulation in the same manner in the validation results (90.3%). The 6 miRNAs that did not match the original observations were without exception up-regulated in lung-cancer, indicating a potential bias of the microarray or qRT-PCR experiments. Applying Fisher's exact test provided evidence for the highly significant validation of the microarray experiments by qRT-PCR, the *p*-value was 4 × 10^−11^. The Top 10 markers that have been successfully validated are provided in Table 1.

**Table 1:** Validated markers NSCLC versus controls

	*t*-test screening	AUC screening	*t*-test validation	AUC validation
**hsa-miR-20b-5p**	1,05E-07	0,07	0,0042071	0,13
**hsa-miR-17-5p**	2,17E-06	0,09	0,00072063	0,13
**hsa-miR-106a-5p**	2,65E-06	0,11	0,00086824	0,13
**hsa-miR-942-5p**	4,04E-06	0,84	0,00011215	0,86
**hsa-miR-20a-5p**	2,01E-05	0,12	0,0034248	0,06
**hsa-miR-29c-5p**	3,67E-05	0,76	1,80E-06	0,92
**hsa-miR-18a-3p**	4,82E-05	0,78	0,001511	0,80
**hsa-miR-378a-5p**	5,37E-05	0,77	5,10E-05	0,85
**hsa-miR-1180-3p**	8,43E-05	0,77	0,046846	0,69
**hsa-miR-126-3p**	9,31E-05	0,17	0,00343	0,07

Beyond the validation of NSCLC versus controls, we also aimed at a validation of markers that differentiate between COPD and NSCLC. Again, we focused on RNU44 as endogenous control and considered raw *p*-values in order to limit a potential bias by different miRNA set sizes. Of the 128 miRNAs that were expressed in the qRT-PCR experiments, 46 were significantly dys-regulated. Of these, 33 were accordingly less- or more abundant in the validation study as well. Although the concordance was not as excellent as for the comparison of unaffected controls versus NSCLC patients, still 71.7% of the markers were successfully validated. Interestingly, we observed similar patterns as in the first comparison. The majority of miRNAs that did not match were up-regulated in the NSCLC patients of the screening cohort. In detail, 13 of the 46 markers were non concordant. Of these, 12 have been up-regulated in the initial screening study in NSCLC patients as compared to COPD patients providing further evidence for a bias in the screening step. The significance value for the validation of the COPD versus NSCLC case was 0.0004 (Fisher's exact test). As for the first comparison, the top 10 miRNAs are displayed in Table 2.

**Table 2:** Validated markers NSCLC versus COPD

	*t*-test screening	AUC screening	*t*-test validation	AUC validation
**hsa-miR-26a-5p**	2,17E-06	0,15	5,53E-05	0,15
**hsa-miR-328-3p**	8,03E-06	0,81	0,00094171	0,74
**hsa-miR-1224-3p**	3,87E-05	0,80	0,0035805	0,72
**hsa-miR-383-5p**	4,29E-05	0,18	0,0011001	0,16
**hsa-miR-93-3p**	0,00010244	0,77	0,0010172	0,75
**hsa-miR-1229-3p**	0,00078496	0,74	1,75E-05	0,84
**hsa-miR-18a-3p**	0,0014703	0,76	7,33E-05	0,78
**hsa-miR-363-3p**	0,0015578	0,22	0,0033026	0,22
**hsa-miR-126-3p**	0,0017084	0,26	0,00097053	0,14
**hsa-miR-199a-3p**	0,0039695	0,26	1,27E-05	0,09

### Comparison between NSCLC, COPD and healthy controls

**Figure 2:**
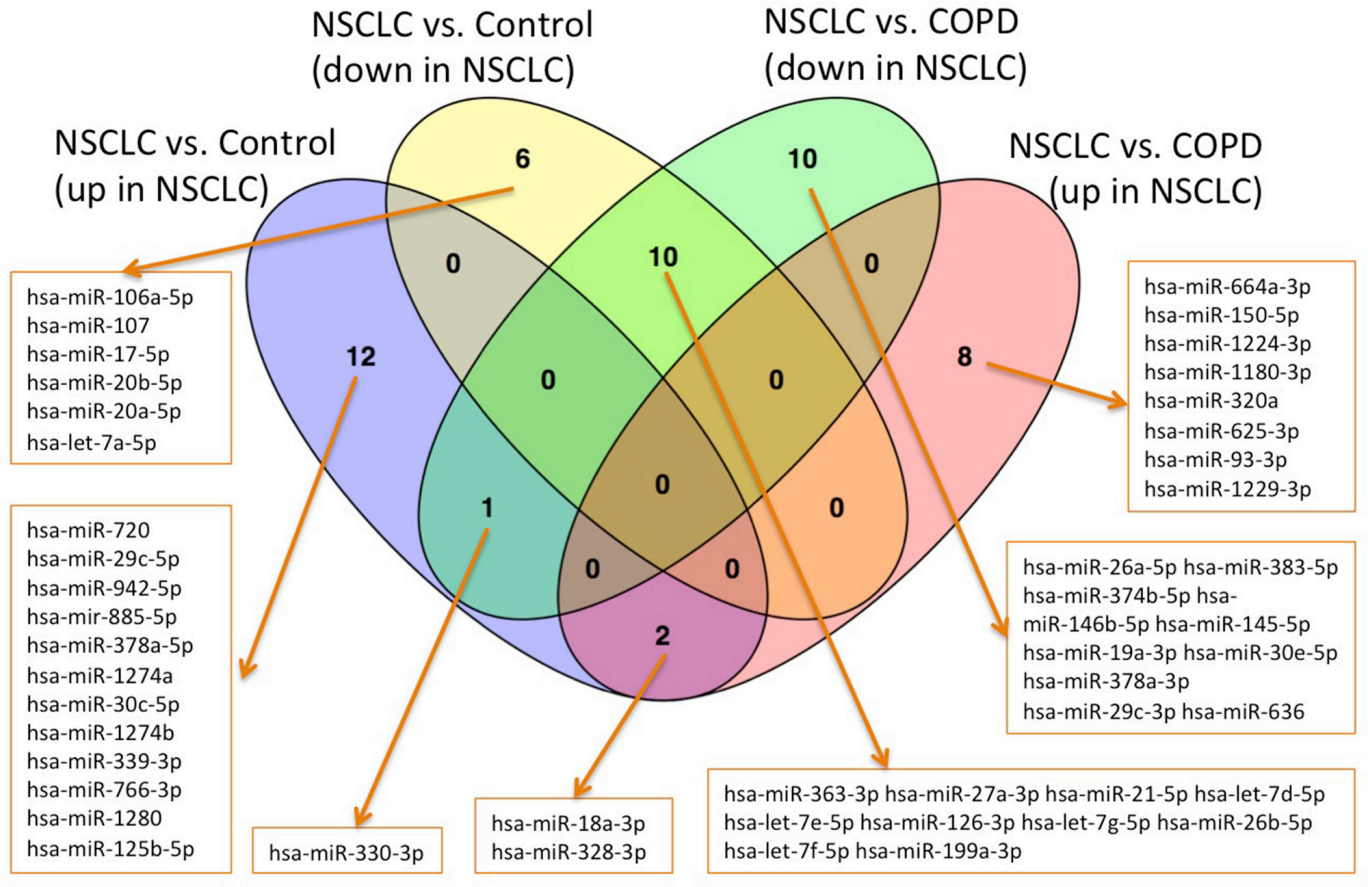
Venn diagrams for the four groups of up- and down-regulated miRNAs in the comparisons NSCLC versus controls and NSCLC versus COPD

**Figure 3:**
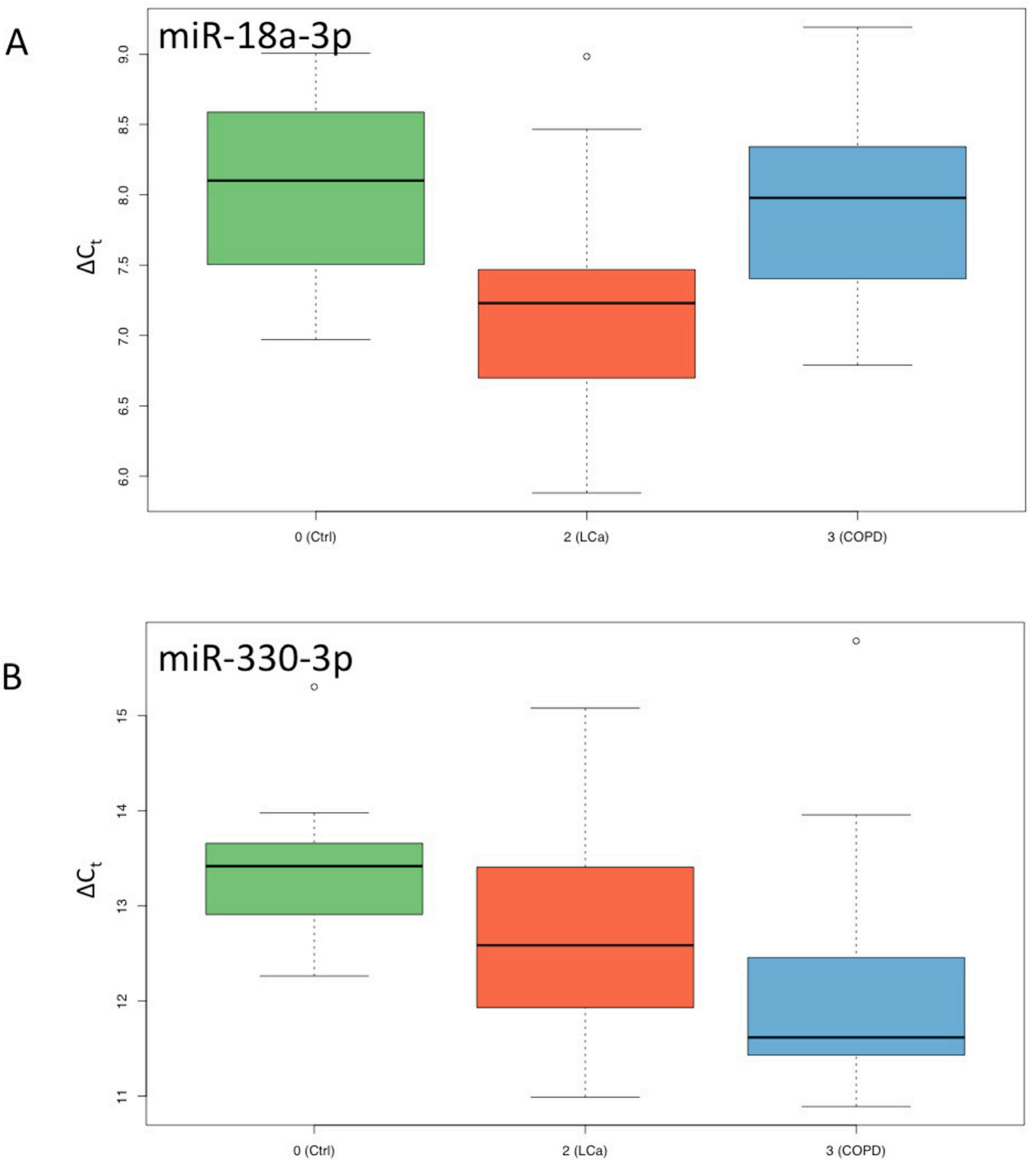
Box-Plots for ΔC_t_ values of miR-18a-3p and miR-330-3p Results for controls are shown in green, for NCSLC in red and for COPD in blue.

We finally used the 128 miRNA panel to compare NSCLC patients, COPD patients and healthy controls. A total of 45 miRNAs was significantly deregulated between NSCLC and healthy controls prior to adjustment for multiple testing, of which 31 remained significant after adjustment. 15 of them were higher abundant in controls and 16 were higher abundant in NSCLC patients. For the comparison of NSCLC versus COPD patients, we observed 46 miRNAs to be significantly altered before and 31 miRNAs remaining after adjustment for multiple testing. 21 of them were higher abundant in COPD patients as compared to the NSCLC patients and 10 were higher in cancer patients. These four miRNA sets are presented in detail in the Venn diagram in Figure 2, which also allows for discovering the overlap between the sets. Largest overlap was computed for miRNAs that are less abundant in NSCLC patients as compared to controls and COPD patients (10 miRNAs). Another two miRNAs were up-regulated in NSCLC patients as compared to controls and COPD patients: hsa-miR-18a-3p and hsa-miR-328-3p. The ΔC_t_ values of miR-18a-3p are exemplarily presented as boxplot for each of the three cohorts in Figure 3A. Finally, one miRNA, namely hsa-miR-330-3p, was up-regulated in NSCLC patients compared to controls but down-regulated in NSCLC patients compared to COPD patients. The ΔC_t_ values of this miRNA for the three cohorts are provided as boxplot in Figure 3B.

We further carried out an analysis of variance (ANOVA) for a three-class comparison between NSCLC patients, COPD patients and healthy controls. In total, 70 of the 128 miRNAs included in the study showed a significant *p*-value prior to the adjustment, of which 63 remained significant following multiple-testing adjustment. The top 10 most significant miRNAs were hsa-miR-199a-3p (*p* = 2 × 10^−9^), hsa-miR-26b-5p (*p* = 6 × 10^−9^), hsa-let-7a-5p (*p* = 2 × 10^−7^), hsa-miR-126-3p (*p* = 3 × 10^−7^), hsa-let-7f-5p (*p* = 6 × 10^−7^), hsa-let-7g-5p (*p* = 7 × 10^−7^), hsa-miR-720 (*p* = 2 × 10^−6^), hsa-let-7d-5p (*p* = 4 × 10^−6^), hsa-let-7e-5p (*p* = 4 × 10^−6^) and hsa-miR-27a-3p (*p* = 5 × 10^−6^). These 10 markers contained 5 members of the let-7 family. Without exception, all of these miRNAs showed the highest ΔC_t_ values in NSCLC patient samples, i.e., lowest expression in NSCLC.

### Classification between NSCLC, COPD and healthy controls using miRNA subsets

**Figure 4:**
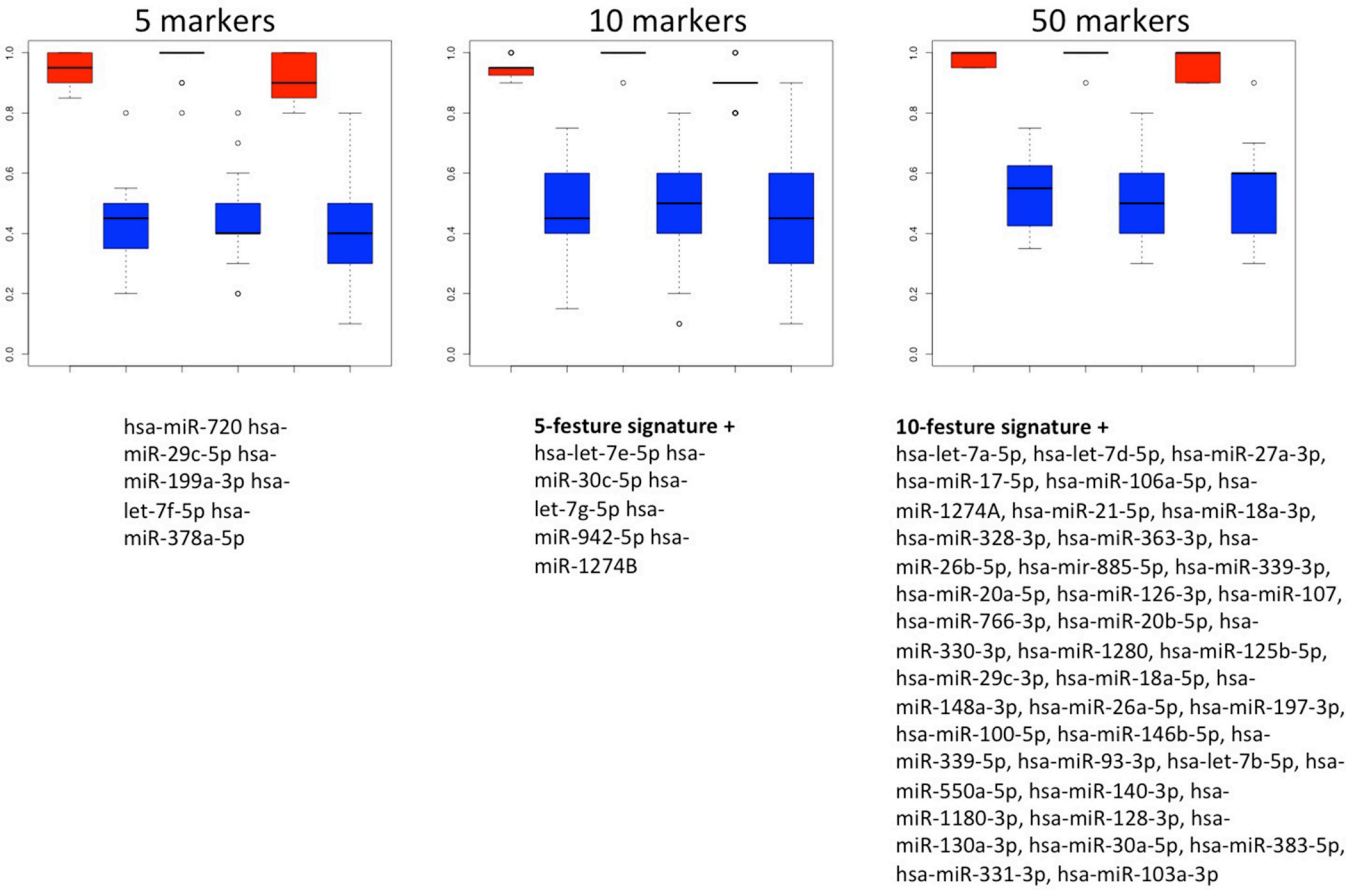
Results of the classification of NSCLC patients vs. healthy individuals Box plots in red show classifications in lung cancer and controls by signatures that use either 5, 10 or 50 miRNAs. The classification results by permutation, i.e., random class labels are shown in blue. Below the boxes the respective marker panels are provided.

To utilize the combined information content of the miRNAs obtained by qRT-PCR toward a multivariate diagnostic signature, we calculated support vector machines with radial basis function kernels. The classification procedure was carried out with subsets of miRNAs in 10 repeated 10-fold cross validations. We performed the calculations also with random class labels to detect potential overtraining. A subset of just five miRNAs was sufficient to reach accuracy of 94.5% (95% CI: 92.5%–96.5%), specificity of 98% (95% CI: 95.7%–100%) and sensitivity of 91% (95% CI: 87.5%–94.5%) in separating NSCLC from control samples. The AUC was 0.978. In increasing the number of features we also computed slightly higher performance rates. For 10 markers the AUC increased to 0.988 at the same averaged accuracy and for 50 markers AUC increased to 0.993 at an accuracy of 98% (specificity 99.5% and sensitivity 96.5%). The classification accuracy, specificity and sensitivity for the 5- 10- and 50 marker set along with the results of the non-parametric permutation tests are presented in Figure 4. Below the respective plots the corresponding signatures are provided.

**Figure 5:**
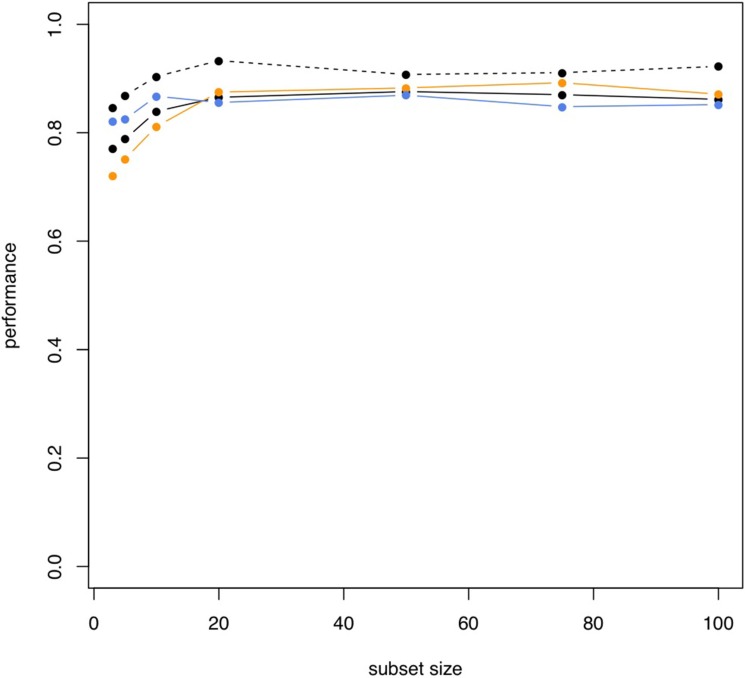
Increasing performance of classification NSCLC versus COPD for increasing subset sizes Black solid line: accuracy; orange line: specificity; blue line: sensitivity; black dashed line: AUC.

**Figure 6:**
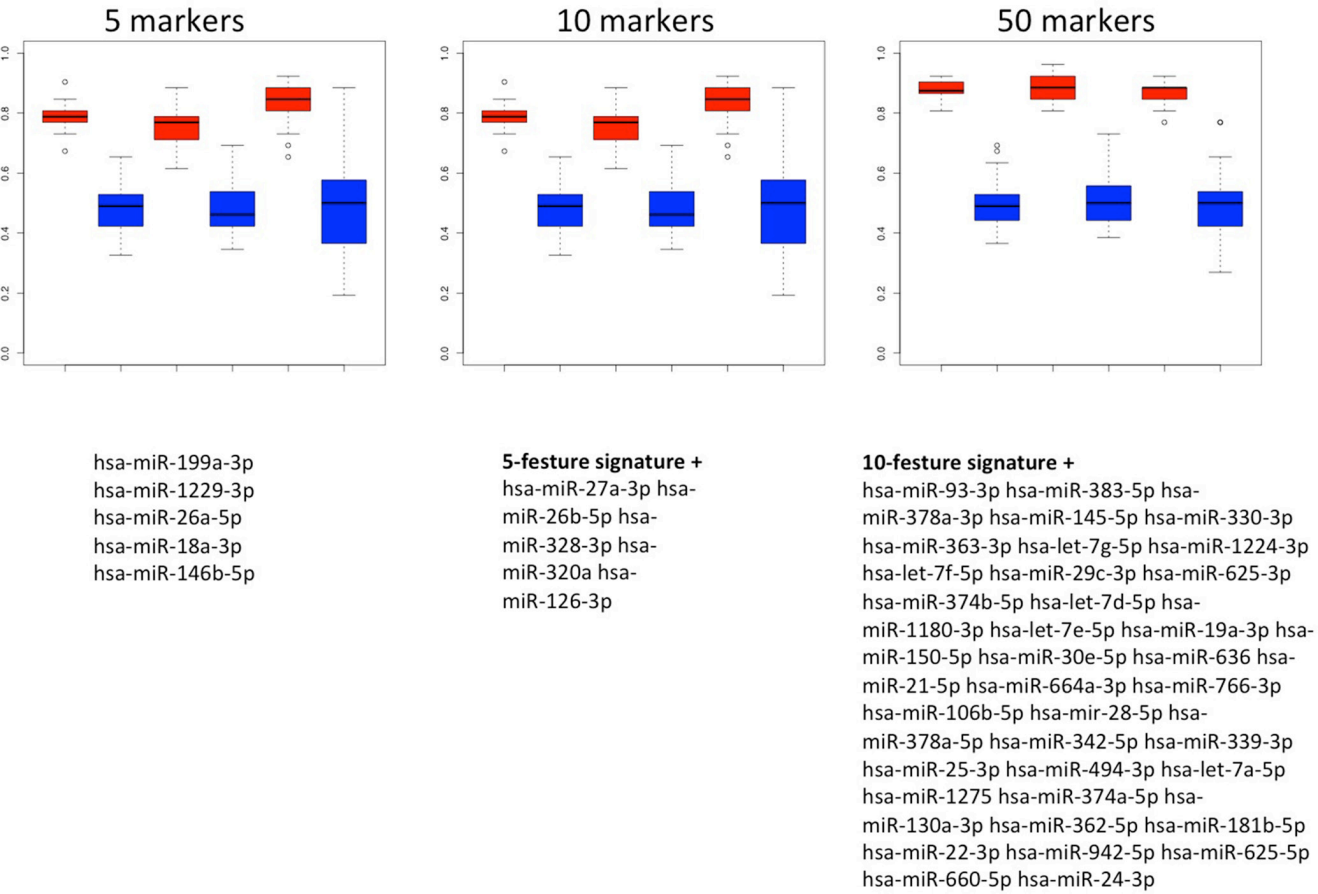
Results of the classification of NSCLC patients vs. COPD patients Box plots in red show classifications in lung cancer and COPD by signatures that use either 5, 10 or 50 miRNAs. The classification results by permutation, i.e., random class labels are shown in blue. Below the boxes the respective marker panels are provided.

We also performed classification in COPD patients and NSCLC patients. In this case, the classification performance was substantially lower compared to the case of NSCLC patients versus unaffected controls. With 5 markers, accuracy, specificity and sensitivity were 78.8%, 75.1% and 82.5%. The AUC in this case was 0.868. While we however just observed a slight increase of performance depending on the number of markers for the first comparison presented above, we here report a rapidly increasing accuracy for larger miRNA sets. The accuracy, specificity, sensitivity and AUC for increasing miRNA marker sets is presented in Figure 5. Using 10 markers, the respective performance criteria increase to 83.9% accuracy, 81.1% specificity, 86.7% sensitivity and AUC of 0.904. For 50 markers, even higher performance was reached: 87.6% accuracy, 88.3% specificity, 86.9% sensitivity and AUC of 0.907. Similar to the calculations for separating NSCLC from controls, the different marker sets with performance metrics for classification in NSCLC versus COPD are presented in Figure 6.

## DISCUSSION

The diagnostic potential of circulating miRNAs is increasingly recognized. In 2008 Chen et al. were first to investigate circulating miRNAs in serum derived from lung cancer patients [15]. Subsequently several studies reported circulating miRNAs in serum or plasma of patients with lung cancer [16–18]. In the present study, we identified hsa-let-7a as one of the miRNAs that essentially contributes to a lung cancer miRNA signature. Differential expression of hsa-let-7a in venous blood from lung cancer patients as compared to tissue and cell lines was already previously reported [19].

In general, complex patterns of miRNAs provide more robust information on a disease status than single miRNAs. Previously we provided first evidence for a blood-based signature that discriminated between tumor patients and healthy controls with an accuracy of 95.4% using microarray data [11, 20]. Subsequently, we showed that blood based miRNAs allow also to discriminate between lung cancer patients, COPD patients, and healthy controls. The comparisons showed that the separation between lung cancer patients and COPD patients is by far more challenging than the separation between lung cancer patients and controls. While 140 miRNAs were significant for the comparison COPD and controls, 61 miRNAs were significant for the comparison lung cancer and controls, and only 14 miRNAs for the comparison between lung cancer and COPD. Nevertheless, we were able to show that blood miRNA signatures are suitable to distinguish lung cancer from COPD with 90.4% accuracy [11, 20].

MiRNAs can be reliably measured from different sources including solid tissues and body fluids (whole blood, serum, plasma, urine and others) the latter of which offer the possibility for a non-invasive or non-invasive or minimally invasive analysis. The analysis of body fluids, however, limits the option of a functional analysis of miRNAs. Circulating miRNAs in serum or plasma cannot be readily traced back to their cell of origin, which can but does not have to be the tumor cell. In this study we measured miRNA patterns from whole blood collected in PAXGene tubes. As shown in our previous studies the miRNA pattern collected in PAXGene tubes largely derives from blood cells including B-, T-, and NK-cells. The measured miRNA pattern of PAXGene tubes likely represents the host response against the tumor and does not mirror the altered gene regulation in the tumor cell itself.

Despite the still increasing numbers of studies not only on circulating miRNAs but also on signatures of circulating miRNAs, there is still an extremely small number of miRNAs that are introduced into a clinical study. This is mainly attributed to the fact that few studies are focusing on confirming and optimizing previously reported signatures – a necessary prerequisite towards clinical application. In the present study, we evaluated the performance of blood based miRNA signatures by Fluidigm qRT-PCR.

Translation into clinical practice, robust results and successful transfer of findings between different platforms all require appropriate internal and/or spike-in controls. This is especially true for qRT-PCR approaches on body fluid samples.

Spike-in controls, which may be added prior to RNA extraction can support the control process further but may also add noise. In this and other studies, we normalized qRT-PCR expression by proven and widely used constitutively and ubiquitously expressed endogenous genes, often referred to as housekeeping genes. We identified and confirmed best performing endogenous normalizers for the detected microRNA expression levels from a group of cellular small RNAs with different expression levels and from different biological process contexts. Endogenous controls provide the advantage that they normalize for the complete process including extraction, reverse transcription and quantitative PCR plus normalization for potentially varying RNA inputs. Utilization of robust endogenous controls is one of the advantages of the PAXgene Whole Blood approach for translation to clinics.

In our abovementioned study we obtained an accuracy of 95.4% for the separation between patients with lung cancer and controls [11]. Excluding those markers that did not match quality criteria or were not expressed in qRT-PCR, we observed a very high concordance between both studies. 90.3% of the markers were concordant in both studies. Fisher's exact test indicated that the replication was highly significant with a *p*-value of 4 × 10^−11^. Among the top validated markers we found hsa-miR-20b-5p, hsa-miR-17-5p and hsa-miR-106a-5p. The classification performance using 5 markers was already 94.5%. In our study, T1 tumor patients could be equally well detected as T2 tumor patients, indicating that our approach is also reasonable for early detection of tumors.

As for the discrimination between NSCLC and COPD, the present Fluidigm qRT-PCR analysis yielded an AUC of 0.908, an accuracy of 87.6%, a specificity of 88.3% and a sensitivity of 86.9%. Our former microarray based study identified among others hsa-miR-675, hsa-miR-93*, hsa-miR-513b, and hsa-miR-1224-3p as significant for the separation between lung cancer and COPD. The present analysis by Fluidigm qRT-PCR identified hsa-miR-93-3p and hsa-miR-1224-3p within a signature that separates COPD from NSCLC patients. In sum, we found a slightly lower, nonetheless still good concordance between microarray screening and qRT-PCR validation for the comparison of NSCLC versus COPD. 33 of 46 markers were concordant. Among the most concordant markers we found hsa-miR-26a-5p, hsa-miR-328-3p, hsa-miR-1224-3p, hsa-miR-383-5p and hsa-miR-93-3p.

Among all miRNAs, the members of the let-7 family play a very important role in the screening and validation study. Among the top 10 markers in an analysis of variance (ANOVA) we observed 5 members of this well-known miRNA family. Hsa-let-7d was previously found to be highly expressed in blood cells with the highest expression in neutrophils [21]. In a former study, we identified hsa-let-7d as down-regulated in eosinophilic, neutrophilic granulocytes and in monocytes of lung cancer patients [22].

A central question is on the origin of certain miRNAs that are dys-regulated in blood. We thus performed a statistical miRNA enrichment analysis and asked whether significantly dys-regulated miRNAs according to the ANOVA are enriched in specific functional categories. To this end, we applied our miRNA Enrichment and Annotation Tool miEAA (http://www.ccb.uni-saarland.de/mieaa_tool/). According to this analysis, multiple markers are from the lung (miRWalk category lung, adjusted *p*-value 0.018, miRNAs hsa-let-7a-5p; hsa-miR-126-3p; hsa-let-7f-5p; hsa-let-7g-5p; hsa-let-7d-5p; hsa-let-7e-5p; hsa-miR-26a-5p; hsa-miR-21-5p; hsa-miR-20a-5p; hsa-miR-146b-5p). Likewise the lung neoplasms category was enriched (miRWalk category lung neoplasms, adjusted *p*-value 0.03, miRNAs hsa-let-7a-5p; hsa-miR-126-3p; hsa-let-7f-5p; hsa-let-7g-5p; hsa-let-7e-5p; hsa-miR-21-5p; hsa-miR-20a-5p; hsa-miR-30c-5p; hsa-miR-29c-3p; hsa-miR-19a-3p).

## MATERIALS AND METHODS

### Study set-up

We collected 120 individual whole blood samples in PAXgene Blood RNA tubes. PAXgene Blood RNA Tubes contain a reagent that lyses blood cells and immediately stabilizes intracellular RNA to preserve the gene expression profile.

The patient cohort includes 74 NSCLC patients, 26 patients with chronic obstructive pulmonary diseases (COPD) without lung cancer and 20 physiologically unaffected controls. While the unaffected individuals had a mean age of 50 years (+/−24 years) the lung cancer patients were on average 66 years (+/− 9 years) and the COPD patients 68 years (+/− 11 years). Information on the individuals is provided in Supplementary Table 1.

### miRNA extraction

Total RNA using miRNA from PAXgene RNA blood collection tubes was extracted either with the miRNeasy Mini Kit (Qiagen) or the PAXgene Blood miRNA Kit (Qiagen). In Supplementary Table 1 the applied isolation Method is listed. For both extraction methods the PAXgene Blood RNA Tubes were first centrifuged to pellet the samples, which were then washed with RNase free water. For the miRNeasy Mini Kit, the pellet was resuspended in 700 μl Qiazol and subsequently further processed according to manufacturer's instructions. For the PAXgene Blood miRNA Kit, the pellet was resuspended in 350 μl Buffer BM1 and subsequently further processed according to manufacturer's instructions. Quantity and quality of the isolated RNA was assessed using NanoDrop-1000 (Thermo Fischer Scientific) and 2100 Bioanalyzer (Agilent).

### Selection of miRNAs for validation

In previous studies we reported blood-borne miRNA signatures for various diseases with a focus on cancer, including lung cancer [11–13]. In all of these studies, the blood was collected in PAXgene blood RNA collection tubes (Becton Dickinson, New Jersey, USA) and total RNA was isolated using the PAXgene Blood miRNA Kit (Qiagen) or the miRNeasy Mini Kit (Qiagen). Based on our abovementioned studies we defined a panel of 235 miRNAs. Among the key criteria of miRNAs in this panel was their differential abundance in unaffected control samples versus both non-small cell lung cancer (NSCLC) samples and chronic obstructive pulmonary disease (COPD) samples as well as for their discrimination between the two pathologies. Additional criteria were absolute expression level, fold changes between group comparisons, and significance values. The complete list of these miRNAs including miRBase v21 nomenclature, miRBase Accession Number (MIMAT) and mature sequence is provided in Supplementary Table 2.

### Fluidigm qRT-PCR dynamic array layout and measurements

We added to our 235 miRNA panel five commonly used endogenous controls (RNU6B, RNU24, RNU44, RNU48, RPL21) for normalization and an internal process control from *C. elegans*. Quantification of miRNA levels in the blinded PAXgene RNA samples was performed on the Fluidigm Biomark HD system using the 96.96 IFC controller and dynamic arrays (Fluidigm; USA). Each dynamic array carried the same number of representative samples from each of the blinded sample groups to avoid any potential batch effects. Each dynamic array also carried non-template controls (NTC) as well as a PAXgene Standard control sample (Comprehensive Biomarker Center GmbH, Heidelberg) to assess inter-plate variation and to allow for calibration if necessary in addition to normalization to endogenous controls. Furthermore, each plate carried all five endogenous control assays to allow for precise normalization. Each miRNA and each endogenous control was measured in triplicates. Reverse Transcription and qRT-PCR reaction were carried out according to the manufacturer's instructions (Fluidigm, USA). In brief, RNA was reverse transcribed using pools of TaqMan RT primers and respective reagents (Thermo Fisher Scientific). Resulting cDNA was pre-amplified in Specific Target Amplification (STA). STA were prepared according to Fluidigm protocols. In brief, STA allows for a multiplexed preamplification of up to 100 targets by using a 0.2X pool of gene expression assays (TaqMan^®^ PreAmp Master Mix and TaqMan Gene ExpressionAssays, both from Applied Biosystems) as the source of primers. STA Amplification products were diluted, loaded onto the 96.96 Dynamic Array Chips for Gene Expression (Fluidigm, USA), each of which allows for the simultaneous microfluidic measurement of 96 sample wells with 96 assays, and subjected to qRT-PCR on the Biomark HD (Fluidigm, USA).

### Biostatistical evaluation

For each miRNA and each sample the three individual measurements have been extracted. Values, which were out of the linear range of detection of 25 C_t_ cycles or failed the internal Quality Score threshold of 0.65, were omitted from the analysis. The Quality Threshold in the BioMark™ Analysis software is a qualitative tool designed to measure the “quality” of each amplification curve. Basically, each amplification curve is compared to an ideal exponential curve and as the quality score approaches 1 the closer it is to ideal. The further the curve is from ideal, its quality score approaches 0. From all other replicates, the median has been calculated as final measurement. When none of the three replicates passed the above criteria, the miRNA for this patient was set to NA. All miRNAs with more than 10 NA values were omitted, for all other miRNAs, the global average measurement for this miRNA has been calculated and NAs have been replaced with the respective estimate. The respective results for each miRNA and patient represent the final C_t_ values that have been stored in a matrix.

To identify the most appropriate endogenous control out of the five measured, we first considered the mean and standard deviation of each control and calculated the coefficient of variation. Additionally, we applied the program NormFinder to select the best endogenous control [14].

For basic biostatistical evaluation in the case of pair-wise group comparisons, *t*-tests have been performed. Since not all data were normally distributed, additional Wilcoxon Mann-Whitney *p*-values were calculated. All *p*-values were adjusted with respect to the false discovery rate by using Benjamini-Hochberg adjustment. Besides significance values the Area Under The Receiver Operating Characteristics Curve (AUC) value was computed. For the comparison of the three groups, analysis of variance has been performed. As further unsupervised statistical approaches, hierarchical clustering has been carried out as well as principal component analysis. For supervised analysis, radial basis function support vector machines were used. These have been evaluated by 10 independent repetitions of 10-fold cross validation. As subset selection technique a filter based on the significance of miRNAs has been applied in a stepwise-forward manner.

## CONCLUSIONS

MiRNA profiles from body fluids have frequently been proposed as novel powerful biomarker candidates. The successful translation of initial screening results into clinical practice requires reproduction on an independent platform and on an independent cohort. We here report miRNA signatures differentiating with high accuracy between lung cancer and unaffected controls and between COPD and lung cancer. These results are consistent with the results of our former microarray studies, and provide further evidence that blood based miRNA signatures are suitable for lung cancer diagnosis including the differentiation between NSCLC patients and COPD patients.

## SUPPLEMENTARY MATERIALS TABLES





## References

[1] Torre LA, Bray F, Siegel RL, Ferlay J, Lortet-Tieulent J, Jemal A (2015). Global cancer statistics, 2012. CA Cancer J Clin.

[2] Bartel DP (2009). MicroRNAs: target recognition and regulatory functions. Cell.

[3] Qin X, Xu H, Gong W, Deng W (2014). The Tumor Cytosol miRNAs, Fluid miRNAs, and Exosome miRNAs in Lung Cancer. Front Oncol.

[4] Joerger M, Baty F, Fruh M, Droege C, Stahel RA, Betticher DC, von Moos R, Ochsenbein A, Pless M, Gautschi O, Rothschild S, Brauchli P, Klingbiel D (2014). Circulating microRNA profiling in patients with advanced non-squamous NSCLC receiving bevacizumab/erlotinib followed by platinum-based chemotherapy at progression (SAKK 19/05). Lung Cancer.

[5] Keller A, Leidinger P, Steinmeyer F, Stahler C, Franke A, Hemmrich-Stanisak G, Kappel A, Wright I, Dorr J, Paul F, Diem R, Tocariu-Krick B, Meder B (2014). Comprehensive analysis of microRNA profiles in multiple sclerosis including next-generation sequencing. Mult Scler.

[6] Leidinger P, Backes C, Blatt M, Keller A, Huwer H, Lepper P, Bals R, Meese E (2014). The blood-borne miRNA signature of lung cancer patients is independent of histology but influenced by metastases. Mol Cancer.

[7] Keller A, Leidinger P, Vogel B, Backes C, ElSharawy A, Galata V, Müller S, Marquart S, Schrauder M, Strick R, Bauer A, Wischhusen J, Beier M (2014). miRNAs can be generally associated with human pathologies as exemplified for miR-144. BMC Med.

[8] Leidinger P, Backes C, Deutscher S, Schmitt K, Mueller SC, Frese K, Haas J, Ruprecht K, Paul F, Stahler C, Lang CJ, Meder B, Bartfai T (2013). A blood based 12-miRNA signature of Alzheimer disease patients. Genome biology.

[9] Margue C, Reinsbach S, Philippidou D, Beaume N, Walters C, Schneider JG, Nashan D, Behrmann I, Kreis S (2015). Comparison of a healthy miRNome with melanoma patient miRNomes: are microRNAs suitable serum biomarkers for cancer?. Oncotarget.

[10] Ma J, Lin Y, Zhan M, Mann DL, Stass SA, Jiang F (2015). Differential miRNA expressions in peripheral blood mononuclear cells for diagnosis of lung cancer. Lab Invest.

[11] Keller A, Leidinger P, Borries A, Wendschlag A, Wucherpfennig F, Scheffler M, Huwer H, Lenhof HP, Meese E (2009). miRNAs in lung cancer - studying complex fingerprints in patient's blood cells by microarray experiments. BMC cancer.

[12] Keller A, Leidinger P, Bauer A, Elsharawy A, Haas J, Backes C, Wendschlag A, Giese N, Tjaden C, Ott K, Werner J, Hackert T, Ruprecht K (2011). Toward the blood-borne miRNome of human diseases. Nature methods.

[13] Leidinger P, Keller A, Borries A, Huwer H, Rohling M, Huebers J, Lenhof H-P, Meese E (2011). Specific peripheral miRNA profiles for distinguishing lung cancer from COPD. Lung Cancer.

[14] Andersen CL, Jensen JL, Orntoft TF (2004). Normalization of real-time quantitative reverse transcription-PCR data: a model-based variance estimation approach to identify genes suited for normalization, applied to bladder and colon cancer data sets. Cancer Res.

[15] Chen X, Ba Y, Ma L, Cai X, Yin Y, Wang K, Guo J, Zhang Y, Chen J, Guo X, Li Q, Li X, Wang W (2008). Characterization of microRNAs in serum: a novel class of biomarkers for diagnosis of cancer and other diseases. Cell research.

[16] Foss KM, Sima C, Ugolini D, Neri M, Allen KE, Weiss GJ (2011). miR-1254 and miR-574-5p: serum-based microRNA biomarkers for early-stage non-small cell lung cancer. Journal of thoracic oncology.

[17] Markou A, Sourvinou I, Vorkas PA, Yousef GM, Lianidou E (2013). Clinical evaluation of microRNA expression profiling in non small cell lung cancer. Lung cancer.

[18] Shen J, Todd NW, Zhang H, Yu L, Lingxiao X, Mei Y, Guarnera M, Liao J, Chou A, Lu CL, Jiang Z, Fang H, Katz RL (2011). Plasma microRNAs as potential biomarkers for non-small-cell lung cancer. Laboratory investigation.

[19] Jeong HC, Kim EK, Lee JH, Lee JM, Yoo HN, Kim JK (2011). Aberrant expression of let-7a miRNA in the blood of non-small cell lung cancer patients. Molecular medicine reports.

[20] Leidinger P, Keller A, Borries A, Huwer H, Rohling M, Huebers J, Lenhof HP, Meese E (2011). Specific peripheral miRNA profiles for distinguishing lung cancer from COPD. Lung cancer.

[21] Pritchard CC, Kroh E, Wood B, Arroyo JD, Dougherty KJ, Miyaji MM, Tait JF, Tewari M (2012). Blood cell origin of circulating microRNAs: a cautionary note for cancer biomarker studies. Cancer prevention research.

[22] Leidinger P, Backes C, Dahmke IN, Galata V, Huwer H, Stehle I, Bals R, Keller A, Meese E (2014). What makes a blood cell based miRNA expression pattern disease specific?—a miRNome analysis of blood cell subsets in lung cancer patients and healthy controls. Oncotarget.

